# Multiple health behaviours and interest in change among people with a mental health condition: A brief report

**DOI:** 10.1016/j.pmedr.2021.101383

**Published:** 2021-04-18

**Authors:** Kate Bartlem, Lauren Gibson, Caitlin Fehily, Simone Lodge, John Wiggers, Jenny Bowman

**Affiliations:** a School of Psychology, University of Newcastle, Callaghan, Australia; b Hunter New England Population Health, Wallsend, Australia; c School of Medicine and Public Health, University of Newcastle, Callaghan, Australia

**Keywords:** Health behaviours, Co-occurrence, Chronic disease, Mental health, Mental illness

## Abstract

People with a mental illness experience a disproportionate burden of chronic disease morbidity and mortality; contributed to by a higher prevalence of health risk behaviours that increase the risk of chronic disease development. Amongst this population there is little understanding of multiple risk behaviour co-occurrence and client interest in improving multiple such risks. A cross-sectional survey was undertaken with a random selection of 557 clients of 12 community mental health services in Australia. Participants reported their engagement in health risk behaviours (tobacco smoking, harmful alcohol consumption, inadequate fruit and/or vegetable consumption, and inadequate physical activity), and those deemed ‘at risk’ reported whether they were seriously considering improving their risk behaviours. Almost all participants engaged in at least one risk behaviour (96.1%), with 78.4% of participants engaging in two or more risk behaviours. Of those with two risks, the most frequently paired behaviours were inadequate fruit and vegetable consumption and inadequate physical activity (39.7%); of those with three risks the most common combination was inadequate fruit and vegetable consumption, tobacco smoking and harmful alcohol consumption (42.5%). Of those at risk for all behaviours, 68.4% were interested in improving multiple behaviours, and 14% were interested in improving all; with these participants being most frequently interested in changing smoking, nutrition, and physical activity (19.3%), followed by all risk behaviours (14.0%). The findings strengthen the evidence that people with a mental illness are interested in improving their health risk behaviours and indicate a need to address the multiple health risks in this population group.

## Highlights

• 78% engaged in multiple risk behaviours.
• 68% interested in improving multiple behaviours.
• Multiple behaviours need addressing for this population.
• Multi-behavioural interventions should be considered.

## 1 Introduction

People with all types of mental illness experience a reduced life expectancy of between 10 and 30 years (Walker et al., 2015). Among this group, mortality from chronic diseases such as diabetes and cardiovascular disease are up to five times the general population, with 70–80% of the excess mortality experienced arising from such conditions (Lawrence et al., 2013). A large contributor to this inequitable burden is a higher prevalence of modifiable risk behaviours that increase the risk of chronic disease development; including smoking, inadequate physical activity, harmful alcohol consumption and poor nutrition; with people with a mental illness at least twice as likely to engage in these behaviours (Lawrence et al., 2013, Bartlem et al., 2015, Stanley and Laugharne, 2014). Australian guidelines for nutrition, physical activity and alcohol provide recommendations which aim to reduce the likelihood of chronic disease risk (National Health and Medical Research Council, 2013, National Health and Medical Research Council, 2020, Australian Government, 2020).

Having multiple risk behaviours increases all-cause and cause-specific mortality rates (Kvaavik et al., 2010); highlighting the importance of research to examine the prevalence of combinations of risk behaviours. In the general population it is well established that risk behaviours co-occur (Meader et al., 2016, Noble et al., 2015). The limited research undertaken among people with a mental illness suggests engaging in multiple risk behaviours is also common (Prochaska et al., 2014, Chwastiak et al., 2011, Bartlem et al., 2018) and more so than among people without a mental illness (Noble et al., 2015, Chwastiak et al., 2011). Amongst 2,075 Australian inpatients with a range of mental health conditions for instance, 88% were at risk for two or more of the four behavioural risks assessed (tobacco smoking, harmful alcohol consumption, inadequate fruit and/or vegetable consumption, and inadequate physical activity) (Bartlem et al., 2018). A US study exploring co-occurrence of multiple risks for cardiovascular disease among 500,000 veterans found those with a psychiatric diagnosis were more than 2.5 times as likely as those without to have all three assessed risks (obesity, current smoking, no weekly exercise) (Chwastiak et al., 2011). However, these studies were unable to identify the specific combinations of health risk behaviours that people with a mental illness engaged in.

According to theories of behaviour change, such as the ‘Theory of Planned Behaviour’, behavioural intention (or ‘interest in behaviour change’) is an antecedent to behaviour change (Ajzen, 2011). Gaining an understanding of client interest in singular versus multiple risk behaviour change is key for developing intervention approaches and targets for behaviour change among people with a mental illness, particularly given existing debate over whether behaviours should be addressed singularly or simultaneously (Prochaska et al., 2014). While interest in improving individual risk behaviours is reportedly high among people with a mental illness (Bartlem et al., 2015, Prochaska et al., 2014), little research has explored interest in changing multiple risk behaviours in this population. To the authors’ knowledge, just one study has explored participant interest in changing risk combinations (Prochaska et al., 2014). In a US study with 693 inpatient smokers with serious mental illness, almost two-thirds (61%) of participants with health risks reported they were prepared to change more than one in the next 30 days (Prochaska et al., 2014). Pairs of risks for which participants were most frequently prepared to change include stress management and depression prevention practices (67%) and sleep hygiene and depression prevention (62%) (Prochaska et al., 2014).

Given the limited existing research exploring co-occurrence and patterns of multiple risk behaviours, and interest in improving multiple such risks among people with a mental health condition, a study was undertaken to explore:

1) The prevalence and co-occurrence of multiple health risk behaviours among people with a mental illness, and
2) Whether those with multiple risks were interested in improving multiple such risks.

## 2 Methods

### 2.1 Design, setting and participants

A cross-sectional telephone survey was undertaken with a random selection of clients of 12 adult community mental health services in one local health district in New South Wales, Australia. Details of the setting and participants have been described previously (Bartlem et al., 2015). Over 12 months, a random sample of 1,106 eligible clients were selected from the electronic medical records system. These clients were mailed an information statement and phoned by trained telephone interviewers to confirm eligibility. Eligible (18 years or older, attended an appointment in the last 2 weeks, not too unwell to participate) and consenting participants were administered a computer assisted telephone interview. Ethical approval was obtained from the Hunter New England HREC (approval No. 09/06/17/4.03) and the University of Newcastle HREC (approval No. H-2010-1116).

### 2.2 Measures

Survey items were developed based on recommended assessment tools and previous community surveys (Bartlem et al., 2015). Socio-demographic characteristics (including psychiatric diagnoses or medication taken within the last two months) were reported by participants and attained through the electronic medical records system. Participants reported their engagement in risk behaviours (tobacco smoking, alcohol consumption, fruit and vegetable consumption, physical activity) during the month prior to seeing their community mental health service (Bartlem et al., 2015). Risk for each behaviour (at risk/not at risk) was defined in line with Australian national guidelines (Table 1). Participants who were classified as being at risk for a behaviour were asked whether they were seriously thinking about: quitting smoking, eating more vegetables and/or fruit, reducing their alcohol intake, and doing more physical activity (yes, no, don’t know), over the next month.

**Table 1:** Prevalence and patterns of chronic disease risk behaviours among people with a mental illness (2012).

Patterns of engagement in risk behaviours^a^^,^^b^	Overall sample (N = 557)		Sub-samples according to number of risks	
**No risks**	**3.9%**	**22/557**		
**One chronic disease risk**	**17.6%**	**98/557**	**Among those with 1 risk (N** = **98):**	
Tobacco smoking only	2.0%	11/557	11.2%	11/98
Harmful alcohol only	0.9%	5/557	5.1%	5/98
F&V^c^ only	12.6%	70/557	71.4%	70/98
PA^c^ only	2.2%	12/557	12.2%	12/98

**Two chronic disease risks**	**35.7%**	**199/557**	**Among those with 2 risks (N** = **199):**	
Tobacco + Alcohol	2.0%	11/557	5.5%	11/199
Tobacco + F&V	11.7%	65/557	32.7%	65/199
Tobacco + PA	0.9%	5/557	2.5%	5/199
Alcohol + F&V	6.3%	35/557	17.6%	35/199
Alcohol + PA	0.7%	4/557	2.0%	4/199
F&V + PA	14.2%	79/557	39.7%	79/199

**Three chronic disease risks**	**32.5%**	**181/557**	**Among those with 3 risks (N** = **181):**	
Tobacco + Alcohol + F&V	13.8%	77/557	42.5%	77/181
Tobacco + Alcohol + PA	0.7%	4/557	2.2%	4/181
Tobacco + F&V + PA	9.3%	52/557	28.7%	52/181
Alcohol + F&V + PA	8.6%	48/557	26.5%	48/181

**Four chronic disease risks**	**10.2%**	**57/557**		
Tobacco + Alcohol + F&V + PA				

a As per Australian national guidelines for reducing risk at the time of the study, risk was defined as: any reported smoking, consuming less than two serves of fruit, or five serves of vegetables per day, consuming more than two standard alcoholic drinks on a regular drinking day (chronic alcohol risk) or more than four standard drinks on any one occasion (short term alcohol risk), or engaging in less than 30 min of physical activity on at least five days a week.

b Participants who reported ‘Don’t know’ responses for engagement in health risk behaviours were classified as being at-risk; those who reported ‘don’t know’ for considering making changes to their behaviours were classified as not being interested.

c F&V = Fruit and Vegetables; PA = Physical Activity.

### 2.3 Statistical analysis

SAS analysis package (SAS, V9.4) was used to analyse the data. Descriptive statistics were used to examine sociodemographic characteristics, the patterns of engagement in multiple risk behaviours, and consideration of improving multiple risk behaviours. Due to the interest in improving behaviours questions only being asked of participants at risk for each behaviour, frequency analyses of behavioural combinations that participants were interested in improving were restricted to those who reported engaging in all four risk behaviours.

## 3 Results

### 3.1 Participants/sample

Of the 1,106 participants randomly selected, 903 (82%) were able to be contacted, of which 129 were excluded due to ineligibility upon contact (primarily being mentally or physically incapable of responding to survey items, n = 69). Of the eligible clients (n = 774), 557 (72%) consented and completed the interview. Approximately half of the participants were male (n = 262, 47.0%), few reported being of Aboriginal and/or Torres Strait Islander origin (n = 27, 4.9%), and the mean age was 40.6 years (SD 15.2; range 18–85). Of 519 participants who reported a psychiatric diagnosis, the most frequently reported was depression (n = 325, 62.6%), followed by anxiety disorders (n = 204, 39.3%), schizophrenia/other psychotic disorders (n = 163, 31.4%), bi-polar disorder (n = 115, 22.2%), and other mental illness (n = 14, 2.7%; note, participants could elect multiple diagnoses). Half (50.6%) were at risk for smoking (n = 282), 86.7% for inadequate fruit and/or vegetable consumption (n = 483), 43.3% for alcohol consumption (n = 241) and 46.9% inadequate physical activity (n = 261).

### 3.2 Co-occurrence of risk behaviours

From the overall sample, almost all (96.1%) participants engaged in at least one risk behaviour: 17.6% were at risk for just one behaviour; 35.7% for two; 32.5% for three; and ten percent (10.2%) for all four behaviours (Table 1). Among those with more than one risk (78.4%); the most prevalent combinations were inadequate fruit/vegetable intake and physical inactivity (14.2%); followed by smoking, harmful alcohol consumption, and inadequate fruit/vegetable intake (13.8%); smoking and inadequate fruit/vegetable intake (11.7%); and all four behavioural risks (10.2%). Among the sub-sample with two risks (35.7%), the most frequently reported paired behaviours were inadequate fruit and vegetable consumption and inadequate physical activity (39.7%), and inadequate fruit and vegetable consumption and smoking (32.7%). Among the sub-sample with three risks (32.5%), the most common combination was inadequate fruit and vegetable consumption, smoking and harmful alcohol consumption (42.5%).

### 3.3 Interest in improving multiple risk behaviours

Among those with one risk, approximately half (51.7%) were considering changing that behaviour (Table 2). Among those with two risks, one quarter (25.8%) reported they were considering improving both behaviours. Of those with three risks, 41.1% were thinking of improving multiple risks (28.3% were interested in improving two of the three, and 12.8% in all three risks). Of those at risk for all four behaviours, most were considering improving more than one risk behaviour (n = 39, 68.4%): 28.1% were considering improving two behaviours; 26.3% for three behaviours, and 14% were considering improving all four. Amongst those engaging in all four risk behaviours, participants were most likely to report an interest in improving smoking, nutrition and physical activity (19.3%), followed by all 4 risks (14.0%). Overall, of those clients at risk for two or more behaviours (n = 435), 23.9% (104) were not considering improving any behaviours, 38.4% (167) were considering improving only one behaviour, and 37.7% (164) were considering improving multiple risks.

**Table 2:** Prevalence and patterns of interest in improving chronic disease risk behaviours among people with a mental illness with at least one risk behaviour.

Number of risk behaviours^a^	Number of behaviours interested in changing^e^	%	n
**Participants with one risk (N** = **89)**^b^	*M* (SD) = 0.5 (0.5)		
	0	48.3%	43
	1	51.7%	46

**Participants with two risks (N** = **198)**^c^	*M* (SD) = 0.9 (0.8)		
	0	30.8%	61
	1	43.4%	86
	2	25.8%	51

**Participants with three risks (N** = **180)**^d^	*M (SD)* = 1.3 (1.0)		
	0	21.7%	39
	1	37.2%	67
	2	28.3%	51
	3	12.8%	23

**Participants with four risks (N** = **57)**	*M* (SD) = 2.2 (1.2)		
	0	7.0%	4/57
	1 Smoking only Alcohol only Nutrition only Physical activity only	24.6% 7.0% 1.8% 8.8% 7.0%	14/57 4 1 5 4
	2 Smoking and alcohol Smoking and nutrition Smoking and physical activity Alcohol and nutrition Alcohol and physical activity Nutrition and physical activity	28.1% 0 12.3% 8.8% 0 1.8% 5.3%	16/57 0 7 5 0 1 3
	3 Smoking, alcohol, nutrition Smoking, nutrition, PA Smoking, alcohol, PA Alcohol, nutrition, PA	26.3% 3.5% 19.3% 1.8% 1.8%	15/57 2 11 1 1
	4	14.0%	8/57

a 22 participants reported no risks.

b 9 missing responses.

c 1 missing response.

d 1 missing response.

e Limited to participants at risk for each individual behaviour.

## 4 Discussion

To the authors’ knowledge, this is the first study to explore patterns of multiple health risk behaviours, and interest in changing multiple risks among people with a mental illness. There was a high level of risk co-occurrence, with 78% of participants at risk for two or more behaviours. Inadequate fruit and/or vegetable consumption and inadequate physical activity were the most frequently paired behaviours (39.7%), while inadequate fruit and vegetable consumption, smoking and harmful alcohol consumption were most frequent among those with three risks (42.5%). Over one third of those with multiple risks were seriously considering improving more than one behaviour (37.7%).

The high level of risk co-occurrence reported in this paper has been found in general population studies (Meader et al., 2016, Noble et al., 2015). These studies have similarly reported that the co-occurrence of low fruit and vegetable intake and inadequate physical activity is high (Meader et al., 2016), however direct comparison with such studies is difficult due to different combinations of risks assessed, and different measures and statistical techniques used (Meader et al., 2016, Noble et al., 2015, Morris et al., 2016). Similarly, the results cannot be directly compared to the limited number of previous studies exploring risk co-occurrence in people with a mental illness, due to differences in methodology and the risk behaviours examined (Prochaska et al., 2014, Chwastiak et al., 2011). However, the prevalence of individual risks is consistent with other studies (Lawrence et al., 2013, Bartlem et al., 2015, Stanley and Laugharne, 2014), and the co-occurrence results are consistent with the high prevalence of multiple risk behaviour engagement reported previously (Prochaska et al., 2014, Chwastiak et al., 2011).

This study further strengthens the argument that people with a mental illness are interested in improving their risk behaviours (Bartlem et al., 2015, Bartlem et al., 2018), with 26% of those with two risks, 41% of those with three risks, and 68% of those with four risks reporting they were seriously thinking about improving multiple behaviours. The finding that a large proportion of individuals with multiple risk behaviours were considering improving multiple such risks is consistent with findings reported by Prochaska and colleagues in the US (Prochaska et al., 2014). This reinforces the need for all health professionals providing care for people with a mental illness to offer support for behaviour change. Health professionals have reported a belief that their clients with a mental illness are not interested in improving their risk behaviours (Chwastiak et al., 2013), and these concerns have been associated with a reduced likelihood of providing care to support behaviour change (Bartlem et al., 2016). This study supports previous research indicating this belief is unfounded.

Limitations of this study include that the sample size constrained the ability to explore statistical clustering of risk behaviours, and the clustering of behaviours for which participants are interested in improving. Future studies should consider exploring the clustering of risk behaviours and the factors associated with such clusters in a larger sample of people with a mental illness, to gain a greater understanding of the relationships between behaviours in this population. Regardless, this study is the first to explore multiple risk behaviour patterns and interest in change among people with a mental illness and has important implications for the development of risk behaviour interventions among people with a mental illness.

Overall, findings indicate that there is a need to address the multiple health risk behaviours of mental health clients. Further research is required regarding the most acceptable, effective and cost effective approach for addressing this need. Given the high level of multiple risks and the interest of people with a mental illness in addressing multiple behaviours, both simultaneous and sequential multi-behaviour interventions need further exploration. For general population interventions, addressing multiple behaviours has been suggested to be advantageous for a number of reasons, including the potential to be more time, treatment and cost efficient than those addressing single behaviours (Pronk et al., 2004), and that addressing behaviours simultaneously may have synergistic effects so that change in one increases the likelihood of change in another (Paiva et al., 2012). However, little research has directly compared singular and multi-behaviour interventions, or simultaneous or sequential multi-behaviour interventions (Meader et al., 2017), and to our knowledge none has done so among people with a mental illness.

## Funding

This research was undertaken with funding from the 10.13039/501100000925National Health and Medical Research Council (ID No 1016650). Dr Kate Bartlem is supported by a National Health and Medical Research Council Early Career Fellowship (#1142272). The funders had no role in the study design, conduct of the trial, analysis, or dissemination of findings.

## Declaration of Competing Interest

The authors declare that they have no known competing financial interests or personal relationships that could have appeared to influence the work reported in this paper.
